# Comparison of DOFS Attachment Methods for Time-Dependent Strain Sensing

**DOI:** 10.3390/s21206879

**Published:** 2021-10-17

**Authors:** Shaoquan Wang, Erik Sæter, Kaspar Lasn

**Affiliations:** Department of Mechanical and Industrial Engineering, Norwegian University of Science and Technology (NTNU), Richard Birkelands vei 2B, 7491 Trondheim, Norway

**Keywords:** structural health monitoring, optical fiber, attachment methods, 3-D printing, distributed strain sensing

## Abstract

Structural health monitoring (SHM) is a challenge for many industries. Over the last decade, novel strain monitoring methods using optical fibers have been implemented for SHM in aerospace, energy storage, marine, and civil engineering structures. However, the practical attachment of optical fibers (OFs) to the component is still problematic. While monitoring, the amount of substrate strain lost by the OF attachment is often unclear, and difficult to predict under long-term loads. This investigation clarifies how different attachment methods perform under time-dependent loading. Optical fibers are attached on metal, thermoset composite, and thermoplastic substrates for distributed strain sensing. Strains along distributed optical fiber sensors (DOFS) are measured by optical backscatter reflectometry (OBR) and compared to contact extensometer strains under tensile creep loading. The quality of the bondline and its influence on the strain transfer is analyzed. Residual strains and strain fluctuations along the sensor fiber are correlated to the fiber attachment method. Results show that a machine-controlled attachment process (such as in situ 3-D printing) holds great promise for the future as it achieves a highly uniform bondline and provides accurate strain measurements.

## 1. Introduction

Maintaining the integrity of structural components and infrastructures over years of service is a considerable challenge, and many structural health monitoring (SHM) systems have been developed for this purpose. Among those, distributed optical fiber sensors (DOFSs), or optical fiber (OF) sensors in short, hold many advantages over traditional SHM technologies. Notably, the OF measures directly on the component, it has a long service life, a good corrosion resistance, a small size, and it is immune to electromagnetic interference.

However, the integration of OF sensors inside the component, or attaching them on the component surface, is still a challenge for many practical applications [1]. A limited scope literature review identifies basic types of attachment methods [2,3,4,5,6,7,8,9,10,11,12,13,14,15] for fixing the optical fibers, as shown in Table 1. Structural engineering applications (concrete, timber, and steel) tend to adhere the OF directly on the surface by a rigid glue [2], pre-embed the OF in a package filled with rigid glue or soft rubber [5,6,7], or attach specialized optical cables to the component [8]. Similar methods are adopted for polymers and polymer composites [9,10]. In addition, the OF can be embedded directly inside the polymer or composite components during the manufacturing process [11,12,13,14,15]. Polymer matrix surrounding the OF enables the strain transfer and protects the sensor. When the OF is attached on the surface, the geometry and the mechanical properties of the bondline will affect the accuracy of strain measurements. Thin and rigid bonding is necessary for accurate transfer of strain. Non-appropriate attachments can decrease the strain transfer coefficient, add noise, and give false measurements [16]. Adhesives, such as epoxy, cyanoacrylate, polyester, and quartz glue, are used quite arbitrarily on many substrate materials. The low surface energy, however, becomes a challenge for reliable bonding on thermoplastics with these standard adhesives [17].

To date, the experimental work on attachment methods of distributed OF sensors has been very limited. This is the first investigation where attachments for DOFS are compared under time-dependent loading. Spatially and temporally varying strain profiles are compared along the OF attachment bondline. Practical solutions for fixing the OF on metals, thermoset composites, and thermoplastics are experimentally compared. Both crosslinked structural adhesives and un-crosslinked melting/fusion-based attachments are employed for the sensor attachment process. All attachments remain intact throughout the creep test, so any glueline durability aspects are out of the scope of this investigation.

Experimental strains from optical fibers revealed distinct regions of strain disturbances at the ingress/egress parts of the DOFS. These regions are present for all OF attachments, affecting distributed strain analysis, especially if the attachment lengths are short. Practical analysis methods are suggested for estimating the lengths of the disturbed regions. Moreover, residual strains, as created by the attachment process, were easily characterized by the optical fiber self-recording measurements. Strain fluctuations along the OF length were related to specific fiber attachment processes. A novel optical fiber attachment method based on polymer extrusion additive manufacturing showed good performance, achieving uniform and accurate OF strain measurements.

## 2. Materials and Methods

### 2.1. Materials

**Figure 1:**
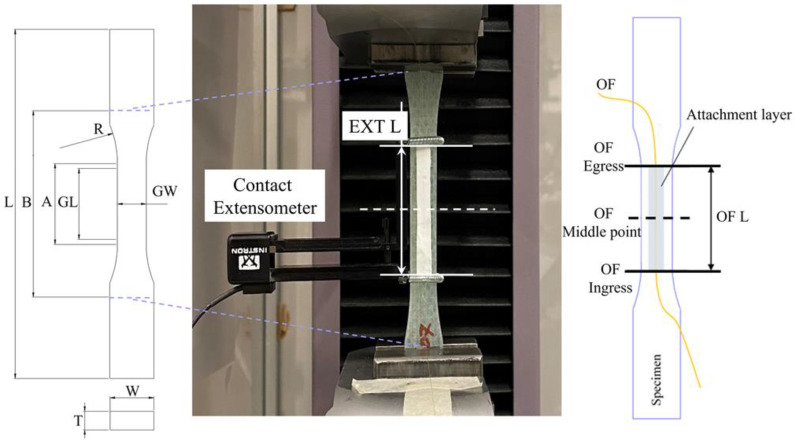
The geometry of specimens and the positioning of the optical fiber (OF).

Three material systems were employed as substrate specimens carrying OFs for tensile creep experiments: a glass fiber epoxy composite, a 3-D-printed PA6 thermoplastic (unreinforced), and a generic mild steel. Between them, a wide range of material behaviors are covered. Structural steel, when loaded within the elastic range, gives negligible time-dependent strains. Unreinforced PA6, on the other hand, creeps extensively already at low loads at room temperature. The GF/Epoxy composite response to creep depends on the lay-up but is somewhere between the previous two materials. Test specimens from all substrate materials were prepared in dogbone shapes, with nominal dimensions adopted from ASTM E8 [18] and ASTM D638 [19] standards, as described in Figure 1 and Table 2.

Steel and GF/Epoxy dogbones were extracted from plates with a water jet cutting system. Steel specimens were cut from a 3-mm-thick plate. The GF/Epoxy plate (7 mm thick) was made by vacuum-assisted resin infusion using 8 layers of 1200 gsm unidirectional 3B HiPer-tex fabric in a quasi-isotropic [90, 45, 0, −45]_s_ layup sequence. The epoxy was mixed from EPIKOTE MGS RIMR 135 and EPIKURE curing agent MGS RIMH 137. PA6 dogbones were built on a PRUSA I3 MK2S 3-D printer from natural Ultrafuse 1.75 mm filaments. The polyamide specimens were also infilled by a [90, 45, 0, −45]_4s_ layup using a 0.2 mm layer height.

All specimens received the same basic surface preparation by cleaning with acetone, abrading with the 120 grit sanding paper, and re-cleaning with acetone, before attaching the OFs. The optical fiber sensor is SMB-E1550H from OFS Fitel. It is a silica/silica/polyimide fiber with a core diameter of 6.5 μm, a cladding diameter of 125 μm, and a coating diameter of 155 μm. Altogether, five attachment methods were used for fixing the optical fibers as summarized in Table 3. These attachment methods were chosen based on previous projects in our lab: a regular cyanoacrylate glue, two types of epoxies (one cold/rapid curing and one hot-curing epoxy film), and two thermoplastic fusion-based attachments (one manual welding and one 3-D printing-based welding). The ‘Embedding’ method by 3-D printing was only employed for PA6 specimens, after being built on the same 3-D printer. All specimens had a single OF installed along the centerline of the specimen, as shown in Figure 1.

### 2.2. Creep Testing

**Figure 2:**
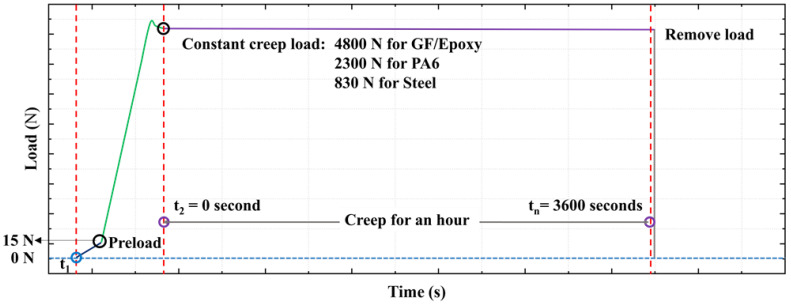
The creep loading procedure.

Mechanical testing was performed on a 5 kN MTS Model 42 universal testing machine. An illustration of the applied creep load–time curve can be seen in Figure 2. At the beginning of the test (time *t*_1_), all specimens were initially loaded to the same preload (15 N), and then further until the defined creep load using a high cross-head speed (100 mm/min for GF/Epoxy and PA6, and 10 mm/min for steel). The small load overshoot before time *t*_2_ is an artifact of control-loop programming. The load adjusted quickly (<40 s) and it was maintained constant with less than 1 N variation during the one-hour creep test. Time *t*_2_ is defined as the start of the creep load, which was kept on until *t_n_* = 3600 s.

### 2.3. Strain Measurements

A contact extensometer Instron 2620-601 with the same gauge length as the OF attachment length OF L in Figure 1, was adopted for independent strain measurements. Extensometer strains are compared to the averaged OF strains of the same specimen.

**Figure 3:**
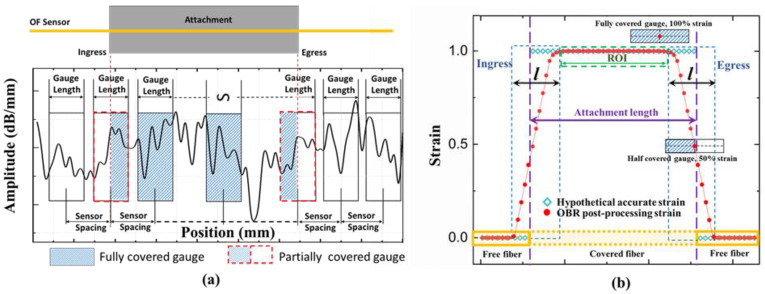
Illustration of OBR post processing parameters (**a**) and strains at the disturbed region *l* at the ingress/egress of the OF (**b**).

Reflectometer OBR 4600 from Luna Instruments was used as the OF interrogator device. Technical details about the entire distributed strain measurement system can be found in Appendix A. To obtain any strain measurement, two light spectra, one from the reference scan and one from the measurement scan, are analyzed in the software. In the program, the OF becomes divided into many virtual strain gauges along the sensing length of the fiber. Each gauge works as a separate virtual strain sensor. All virtual sensors have the same gauge length and spacing between them as shown in Figure 3a. In our work, a sensor spacing of 0.5 mm and a gauge length of 10 mm were selected. This gives overlapping virtual strain gauges. Based on previous experience, this configuration is a good compromise between high spatial resolution and unwanted noise occurrences. Strains are calculated from the frequency shifts of the measured spectrum and averaged along the length of each virtual sensor. Thus, some strains from the ingress and egress parts of the OF can be artificially smaller than the natural strain in the surrounding material. The OBR measured/calculated strain curve in the ingress and egress parts tapers gradually as shown in Figure 3b. The disturbed region *l* contains a gradually increasing curve in the ingress region and a gradually decreasing curve in the egress region. It would seem reasonable to assume that *l* should be equal to the chosen virtual gauge length value. However, the OBR system uses a cross-correlation algorithm to compare the spectra before and after loading. When only a small part of the virtual strain gauge exceeds the attachment length, the calculated strain will not decrease just yet. The disturbed region *l* therefore turns out to be slightly smaller than the gauge length [20]. Avoiding inaccurate ingress/egress regions *l*, only strains from the central region of the attached fiber can be used to calculate the average OF strain. This quasi-constant central region is referred to as the region of interest (ROI) as indicated in Figure 3b.

The OBR measurements were recorded every 60 s throughout the 1 h creep test. In order to calculate strain, three kinds of reference measurements were used. References taken before the OFs were attached on the specimens (i.e., free OFs) are hereby denoted as free-fiber references. Strains calculated by comparing to the free-fiber reference are called *relative free-fiber strains*. Two kinds of references were taken at times *t_x_* of the creep test, where *x* =1, 2, as seen in Figure 2. The strains obtained by comparison to the reference at *t_x_* are called *relative-t_x_ strains*. Relative-*t*_2_ strains represent the time-dependent strain development during tensile creep. Relative-*t*_1_ strains are similar, but additionally include the strain from the load ramp-up procedure.

## 3. Results

Tensile creep testing was carried out on GF/Epoxy, PA6, and steel specimens. Creep loads were applied as 4800, 2300, and 830 N, which acting on 91, 83.2, and 18 mm^2^ cross-section areas, gave approximately 2500, 18000, and 250 με initial (short-term) strains, respectively.

In the current Section 3, raw data from the tests is displayed as follows: (i) experimental strain measurements from three types of substrate specimens are presented by different OF attachment methods, separately; and (ii) spatial and temporal strain curves are accompanied by a coarse analysis of presented data. A more detailed analysis is carried out later in Section 4.

### 3.1. GF/Epoxy Composite

**Figure 4:**
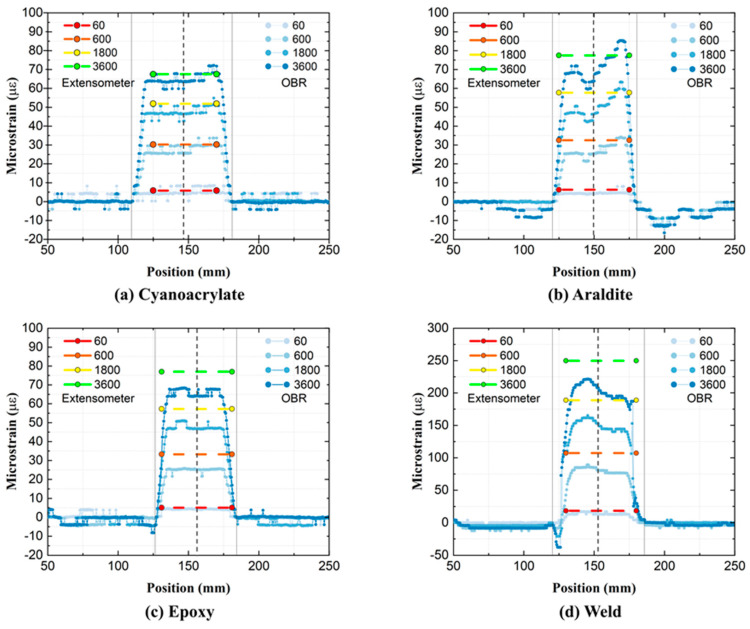
Relative-*t*_2_ spatial strain profiles on GF/Epoxy specimens.

**Figure 5:**
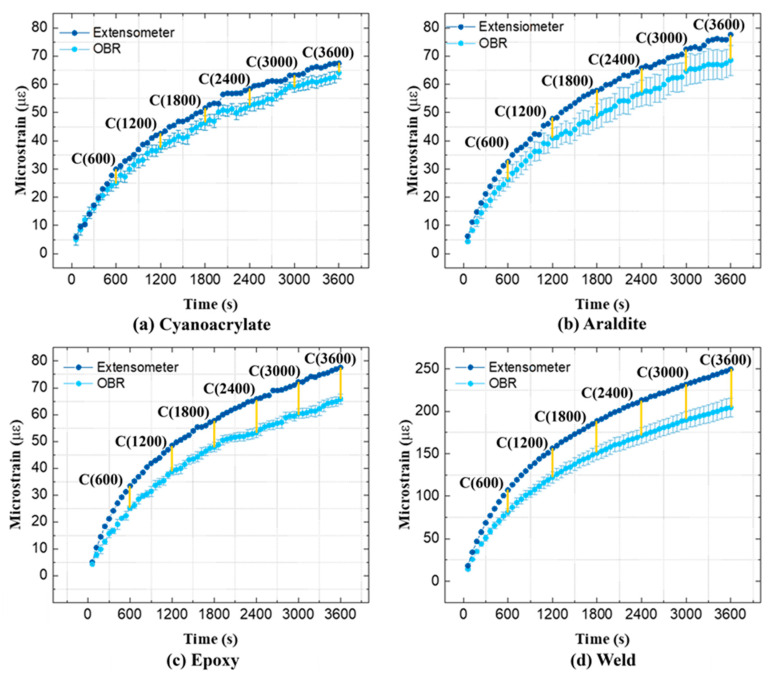
Average relative-*t*_2_ strains of GF/Epoxy specimens measured throughout the creep test.

Figure 4 shows the relative-*t*_2_ spatial strain profiles obtained by the OBR at 60, 600, 1800, and 3600 s after *t*_2_. Analogous relative-t_2_ strains from the contact extensometer, constant within the EXT L gauge length, are also plotted on the same figure. The midpoints and the start/end points of the OF attachments are indicated by vertical dashed and solid lines, respectively. These positions correspond to the same markings in Figure 1. The OF attachment length (OF L) is nominally the same as EXT L of the extensometer; however, some adhesives flowed during the curing process, leading to a longer actual OF L for these attachments. The ROI was defined as 40 mm in the center of the OF L to calculate the average relative-*t*_2_ OF strains shown in Figure 5. The error bars in Figure 5 show ±1 standard deviation for the OF strain profiles within the ROI.

In Figure 4, all OF attachments are seen to behave qualitatively in a similar fashion. The OFs display positive strains in reverse bath-tub profiles, which increase with time during creep loading, as expected. Strains from the ‘Cyanoacrylate’ and ‘Epoxy’ film attachments show a more consistent flat plateau than from the manually applied ‘Araldite’ and ‘Weld’ attachments. However, even the biggest strain fluctuation, e.g., in ‘Araldite’ (ca. 25 με) is small compared to the initial strain from loading the GF/Epoxy specimen (ca. 2500 με). Only the OF strain profile of the ‘Cyanoacrylate’ attachment compares well to corresponding strains from the extensometer. The OF strains from ‘Araldite’, ‘Epoxy’, and especially the ‘Weld’ attachments, are clearly lower than the corresponding extensometer strains.

A detailed temporal comparison of average relative-*t*_2_ strains from the OBR and the extensometer is shown in Figure 5. All strains increase with time, while the slope of the curve decreases. Relative-*t*_2_ extensometer strains behave generally in the same manner as the averaged OF strains during creep. The relative-*t*_2_ strain increase for the ‘Weld’ attachment is ca. 200 με after 3600 s, while it is only around 80 με for all other attachments. Since both the OBR and the extensometer show similarly high values for the ‘Weld’ attachment, this inconsistency must arise from an unknown variability in the specimen production/preparation. Throughout all creep testing, the strains from the OBR are consistently smaller than strains from the extensometer. The difference between the OBR and extensometer strains is smallest for the ‘Cyanoacrylate’ attachment compared to the other three attachments. The yellow markings with coefficients *C* (time) in Figure 5 are clarified and discussed later in Section 4.

### 3.2. Thermoplastic PA6

**Figure 6:**
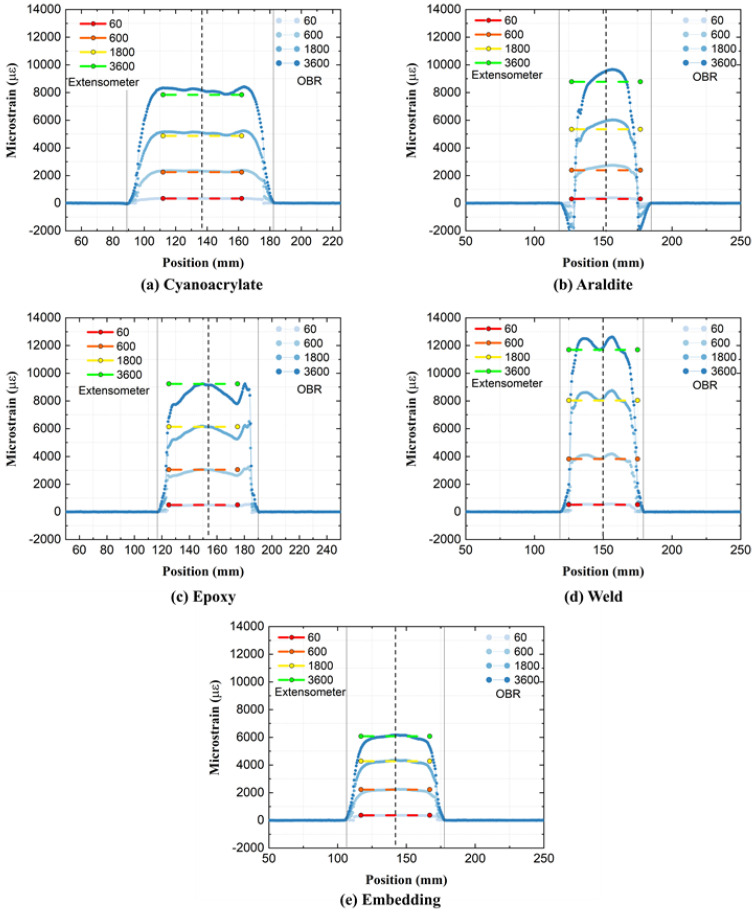
Relative-*t*_2_ spatial strain profiles on PA6 specimens.

**Figure 7:**
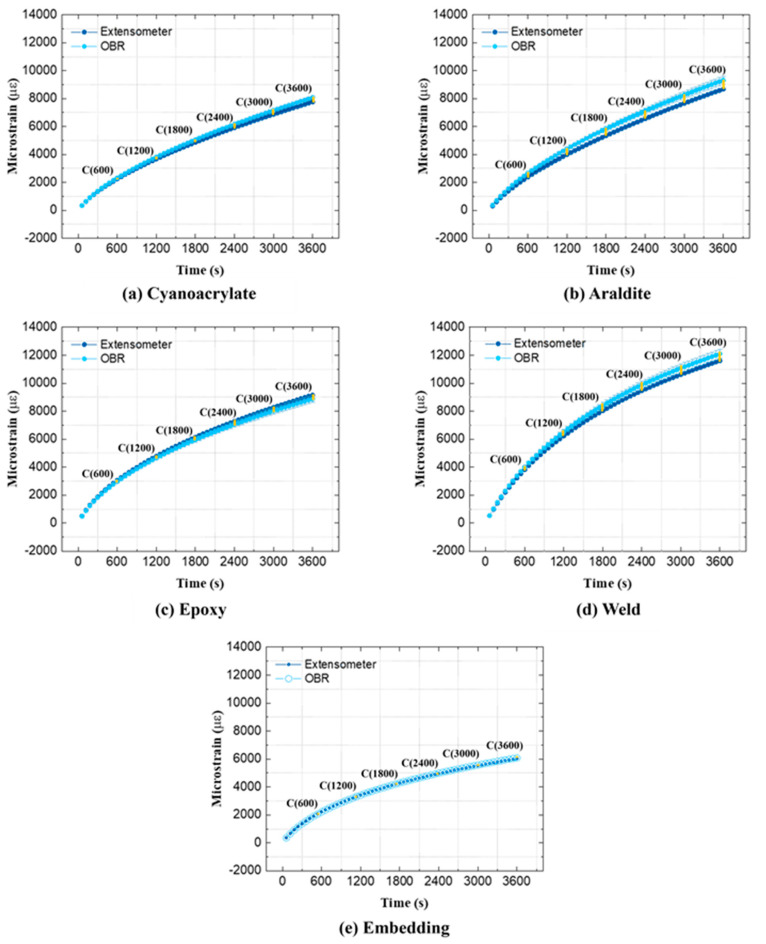
Average relative-*t*_2_ strains of PA6 specimens measured throughout the creep test.

The PA6 specimens were initially loaded to very high strains of ca. 18000 με. Thereafter, Figure 6 shows how relative-*t*_2_ strains along the OFs compare to the corresponding strain profiles from the extensometer. As relative-*t*_2_ strains exceed 6000–12000 με after 1 h of creep, overall strains approaching 3% are hereby measured on PA6 dogbones. Regardless of the high strain values, all attachments display strain curves that are qualitatively similar to the GF/Epoxy specimen curves previously. Strains fluctuate with respect to position and rise with increasing time. The new attachment ‘Embedding’ by 3-D printing also shows similar characteristics to other attachments. Notably, the strain profiles of the ‘Embedding’ attachment are very consistent and uniform. The profiles of ‘Cyanoacrylate’ and ‘Embedding’ attachments compare best to corresponding strains from the extensometer. OF strains from ‘Araldite’ and ‘Weld’ attachments are higher than the corresponding extensometer strains, while OF strains from ‘Epoxy’ are lower. The temporal development of average relative-*t*_2_ strains on the PA6 specimens in Figure 7 was calculated using the same ROI = 40 mm as for GF/Epoxy specimens. For GF/Epoxy, the strains from the OBR were consistently smaller than strains from the extensometer. This relationship is more complex for PA6, as shown in Figure 7, where the OBR strains are now measured larger for ‘Cyanoacrylate’, ‘Araldite’, and ‘Weld’ attachments. For the ‘Embedding’ attachment, excellent agreement between OBR and extensometer strains can be noted.

### 3.3. Steel

**Figure 8:**
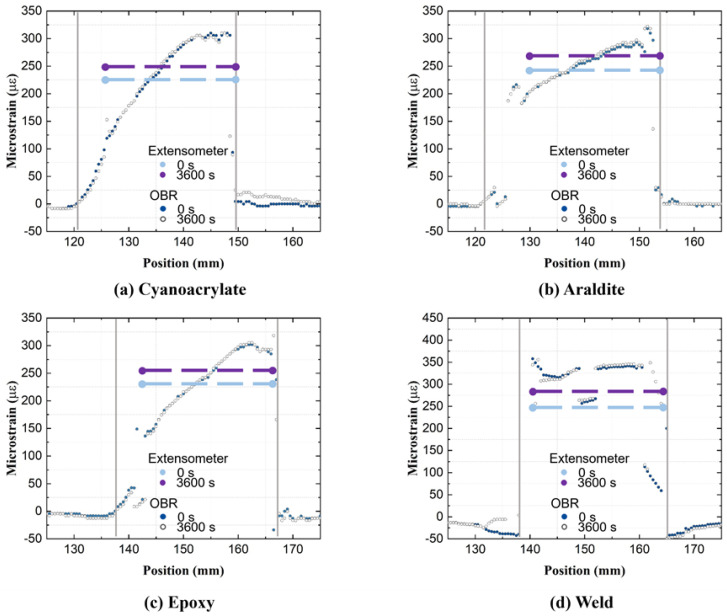
Relative-*t*_1_ spatial strain profiles on steel specimens.

**Figure 9:**
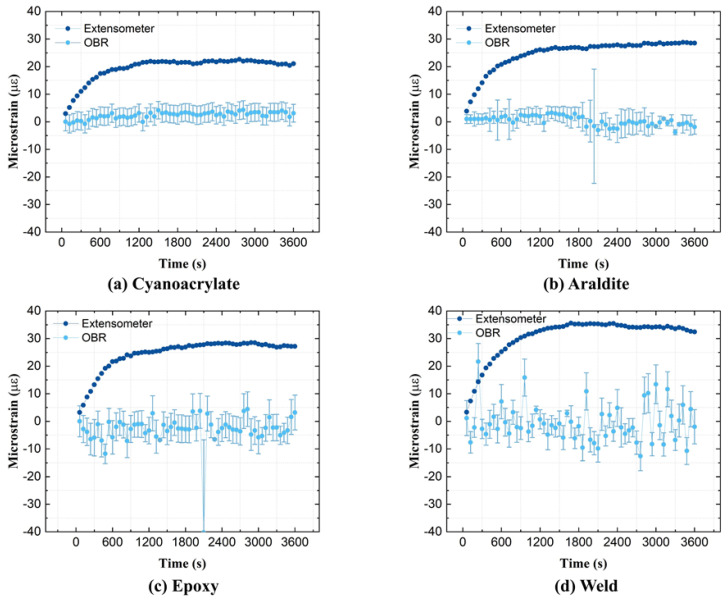
Average relative-*t*_2_ strains of steel specimens measured throughout the creep test.

Creep strains are very small for steel specimens at room temperature. Therefore, relative-*t*_1_ strains, i.e., using reference measurements from unloaded specimens, were chosen for the spatial strain data analysis. Relative-*t*_1_ strain profiles from the OBR and from the extensometer are compared in Figure 8. All strain profiles from the OBR (except for the ‘Weld’) show high variability along the attachment length. This can be caused by the small size of the steel specimen, which negatively affects the practical attachment procedure. It proved difficult to manually handle OFs over short attachment lengths, and to fix them uniformly onto small-sized steel specimens. Expectedly, the OBR strains did not change much during creep loading. However, the extensometer strains appear to increase with time by ca. 20–35 με. This was unexpected; however, it was witnessed from all experiments in Figure 8. In order to clarify how strains from the OBR and the extensometer diverge during creep, average relative-*t*_2_ strain developments (using ROI = 15 mm) are shown in Figure 9. All strain–time curves measured by the OBR fluctuate around zero. Contrary to the OBR, the extensometer shows increasing strain–time curves before ca. 1500–1800 s and then the curves remain flat. This behavior was seen on all specimens consistently, and it is likely related to the warmup drift of the extensometer. Potential issues that can affect contact extensometer strains are briefly summarized in Appendix B.

## 4. Discussion

Based on the raw data from Section 3, the differences between the adopted attachment methods are further analyzed and discussed in detail. In addition, residual strains are presented, and their creation mechanisms are discussed. Correlations between attachment methods and strain data are also emphasized.

### 4.1. The OF Attachment Process

As seen from Figure 4 and Figure 6, different OF attachment methods produce different shapes of spatial strain profiles. Not only are the mean values different, but the strain profile variability along the OF is clearly different. The OF attachment process appears to affect the strain profile variability. Well-controlled attachment methods (‘Cyanoacrylate’, ‘Epoxy’, ‘Embedding’) tend to produce more uniform strain profiles compared to less-controlled methods (‘Araldite’, ‘Weld’). Small imperfections, such as small cracks, thickness variations in the adhesion layer, and misalignment of the attached OF, are well-known quality issues. These imperfections are created in the attachment process, producing noisy datapoints or local distortions in the strain profile.

#### 4.1.1. Residual Strains

**Figure 10:**
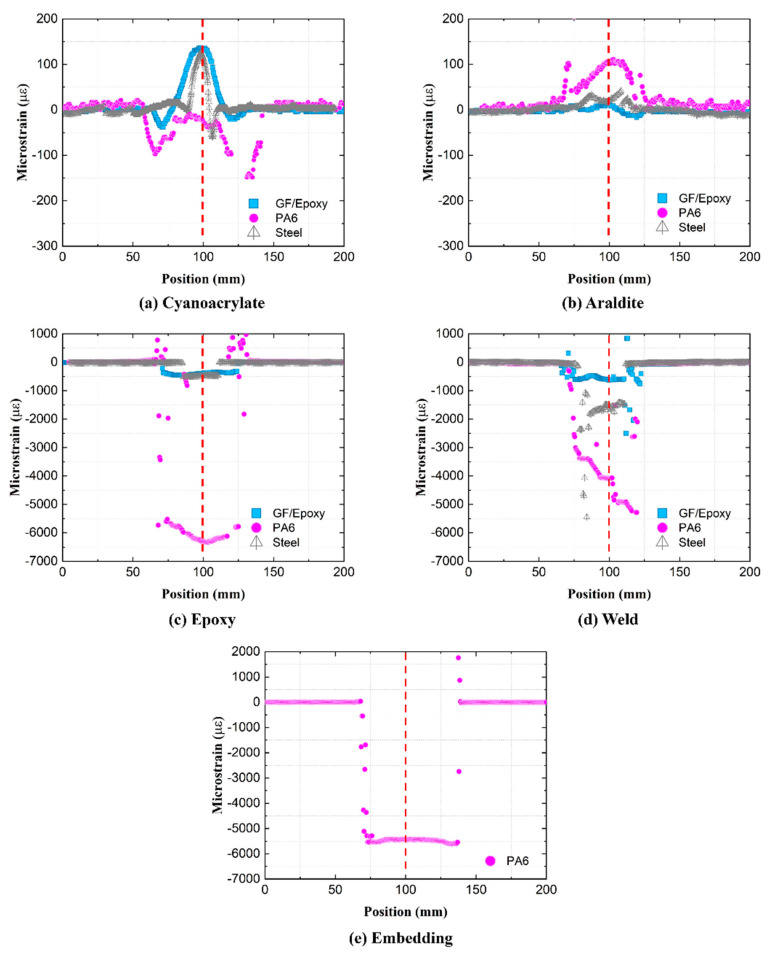
Residual strain profiles along the attached OFs.

Residual strains are created in the attachment process when fixing the OFs to the substrate, before any external mechanical loading occurs. These residual strains are not trivial to predict or measure by conventional means. However, they can be characterized directly, since the OF works as a strain sensor throughout the attachment process. To this end, the pre-attachment free fiber is taken as the reference state, and the load-free condition after the attachment (without any external loading) as the measurement state. Figure 10 shows residual strains from all five attachments on steel, GF/Epoxy, and PA6 substrates. The centerlines of substrate specimens are shifted to a common generic 100 mm coordinate, marked by a vertical dashed line.

**Figure 11:**
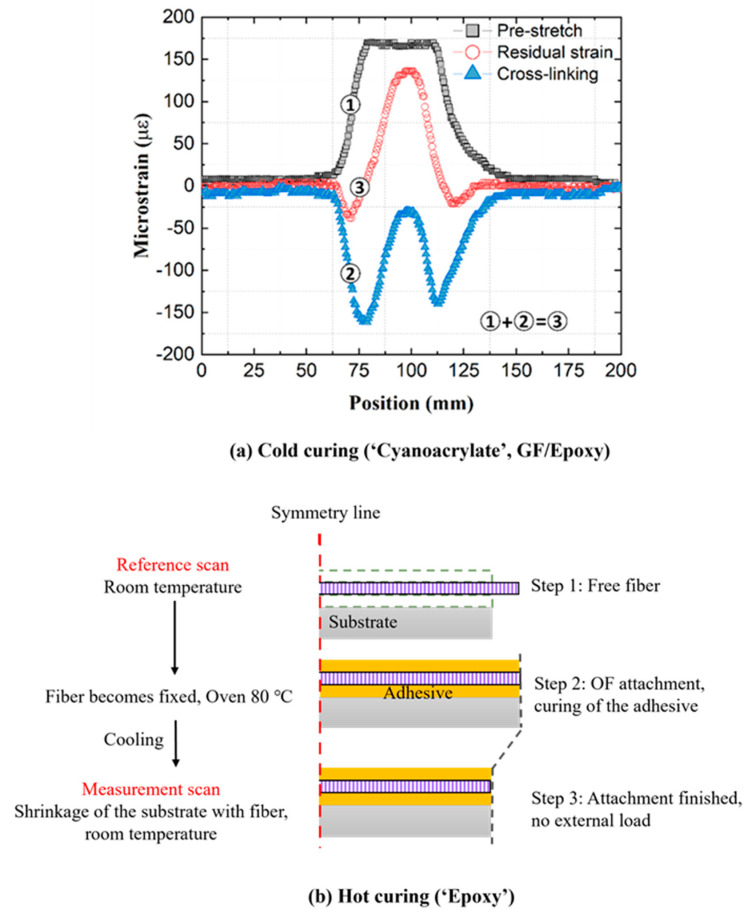
Residual strain formation for cold and hot curing OF attachments.

Residual strains from cold curing ‘Cyanoacrylate’ and ‘Araldite’ attachments from Figure 10a,b are small (below ±150 με) on all substrates. They are created by a combination of compressive shrinkage and a small tensile pre-stretch, applied by hand on the optical fiber. In room-temperature curing, compressive strains are generated from chemical volumetric shrinkage during crosslinking. At the same time, during the installation process, the OF was slightly stretched (using two tapes outside the gauge area) to align it with the specimen. This pre-stretch was hand-controlled, and thus the magnitude of tensile strain varied over different specimens. Figure 11a illustrates in detail how in these cold-curing cases the overall residual strain consists of the pre-stretch (measured during the attachment process) and from the curing shrinkage (calculated by subtracting the pre-stretch from the overall residual strain).

For the ‘Epoxy’ attachment in Figure 10c, residual strains on all specimens are predominantly compressive and much larger than residual strains from the cold curing ‘Cyanoacrylate’ and ‘Araldite’ attachments previously. During the fiber attachment, the specimens were heated in an oven for 12 h at 80 °C to cross-link the epoxy film and then cooled back down to room temperature. In addition to chemical volumetric shrinkage of epoxy from curing, the residual strains of the ‘Epoxy’ attachment originate from the physical volumetric shrinkage of substrates during the cooling process. This process is illustrated in Figure 11b. The temperature change for all three specimens is ca. 60 °C, while the CTE of PA6 (80–90 μm/(m°C)) is much larger than CTE of steel (9–17 μm/(m°C)), and CTE of quasi-isotropic GF/Epoxy (12–20 μm/(m°C)). Thus, residual strains on PA6 became much larger (ca. −6000 με) than residual strains on GF/Epoxy and steel specimens (ca. −500 με).

Residual strains for the manually applied ‘Weld’ attachment in Figure 10d appear less uniform. The mechanism of residual strain creation becomes rather complex, as the strain value is affected by the local shrinkage of PA6 filament (after hot-air welding), and similarly, local contraction of substrates during cooling. Because of locally inconsistent temperatures from the hand-controlled weld process, uneven distributions of residual strains along the OFs are created.

The residual strains of in situ ‘Embedding’ (Figure 10e) originate from the shrinkage of cooling from the deposited PA6 filament during the 3-D printing process [15]. Printing parameters, such as the temperature, extrusion speed, and printing speed, are automatically well controlled, resulting in a high but very uniform residual strain distribution along the attached OF.

In summary, the residual strains of ‘Cyanoacrylate’, ‘Araldite’, and ‘Weld’ attachments are strongly affected by local effects in the attachments process. Thereby, residual strain becomes very inconsistent. In contrast, the residual strains of the ‘Epoxy’ and ‘Embedding’ attachments originate from a global and more uniform source of strain on the specimens. Fluctuations in the residual strain profile refer to a non-uniform occurrence in the bondline in terms of thickness, small cracks, etc. The nonuniform cooling process of the specimen (even for ‘Epoxy’ when taken out of the oven) may also contribute to some variations in residual strains.

#### 4.1.2. Correlation between Residual Strains and Creep Strains

**Figure 12:**
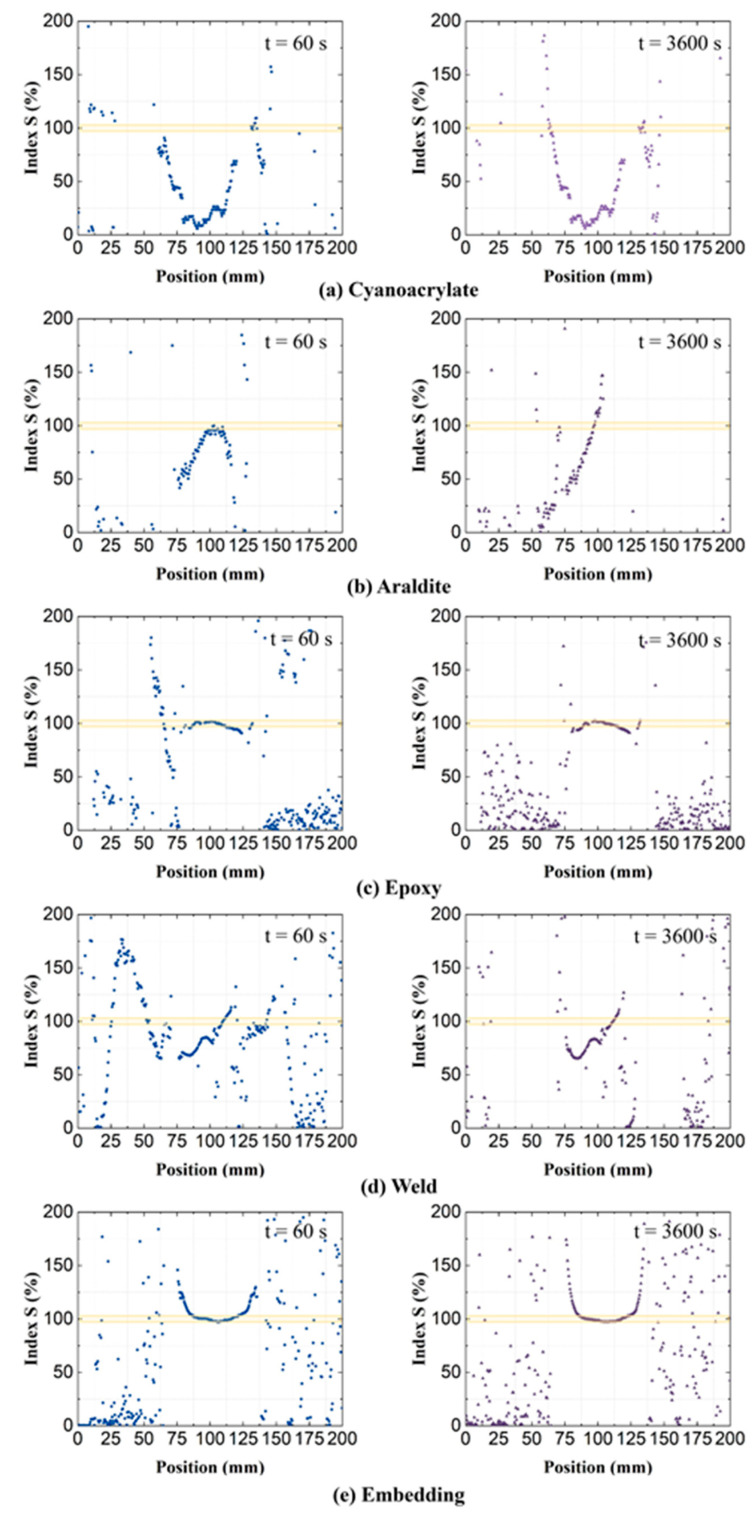
Index *S*, the quotient of normalized residual strains and normalized relative-*t*_2_ creep strains, on PA6 substrates.

During data analysis, correlations between the residual strains and creep strains were noted for some attachment methods. To visualize these correlations, residual strains and relative-*t*_2_ OF strains (at 60 and 3600 s) were first normalized by the peak values of strain curves within the ROI. Then, correlations between the normalized residual strain and normalized relative-*t*_2_ strain were visualized by calculating an index *S*:

(1) $$S(t)=\left|\frac{\varepsilon_{R}^{N}(t)}{\varepsilon_{t_{2}}^{N}(t)}\right|\times 100\%$$

where $\varepsilon_{R}^{N}$ is the normalized residual strain and $\varepsilon_{t_{2}}^{N}$ is the normalized relative-*t*_2_ strain. Figure 12 shows the calculated indices *S*(*t*), for all attachment types on PA6 specimens, at *t* = 60 s and *t* = 3600 s. Specifically, from Figure 12c,e, it is easy to see excellent, nearly one to one correlation between the normalized ‘Epoxy’ and ‘Embedding’ strains between 80 mm and 130 mm position along the attachment length, while the *S* index varies randomly elsewhere. This correlation shows how creep strains that develop later in life are affected by the specific (imperfect) attachment process.

Clear correlations between residual strains and creep strains were only witnessed for the ‘Epoxy’ and ‘Embedding’ attachments. As discussed in Section 4.1.1, the residual strains of ‘Cyanoacrylate’, ‘Araldite’, and ‘Weld’ attachments are heavily affected by local (thermal) effects in the attachment process. Fluctuations in their residual strain profiles are more random due to these local variations.

#### 4.1.3. Variability of Creep Strains

**Figure 13:**
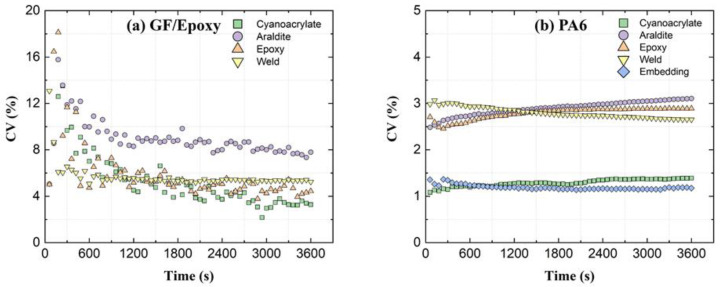
OF strain variability expressed by the coefficient of variation (CV) vs. time for different attachment methods.

As seen from the strain data presented in Section 3, all fiber attachment methods produce somewhat nonuniform spatial strains along the OF sensor length. A nonuniform bondline induces fluctuations in the profiles of the measured strains. Figure 13 compares the coefficient of variation (CV) from different attachment methods. These CVs are calculated from spatial relative-*t*_2_ OF strains (SD can be seen as error bars in Figure 5 and Figure 7). Well-controlled attachment methods (e.g., ‘Cyanoacrylate’, by virtue of low viscosity) show lower variability than hand-controlled and more viscous attachments (‘Araldite’ and ‘Weld’). Machine-controlled 3-D printed ‘Embedding’ attachment has the lowest strain variability. The initial CV of GF/Epoxy in Figure 13a shows very large values compared to the CV obtained later in the creep test. As a ratio (SD divided by the mean), CV is affected by the variations in SD as well as in the average value. Specifically, when the average value is very small, a situation similar to division by zero is approached. As seen in Figure 5 and Figure 7, the initial average strain of the GF/Epoxy specimens is ca. 5 µε, while it is ca. 340 µε in PA6 specimens. Apart from these initial high CV values, Figure 13a,b show that CV of the OF spatial strain profile remains nearly constant through the 1 h creep test. This means, SD increases in constant proportion to the mean for all tested attachment types.

### 4.2. The Accuracy of OBR Strains

#### 4.2.1. The Choice of ROI (and Disturbed End Regions)

**Figure 14:**
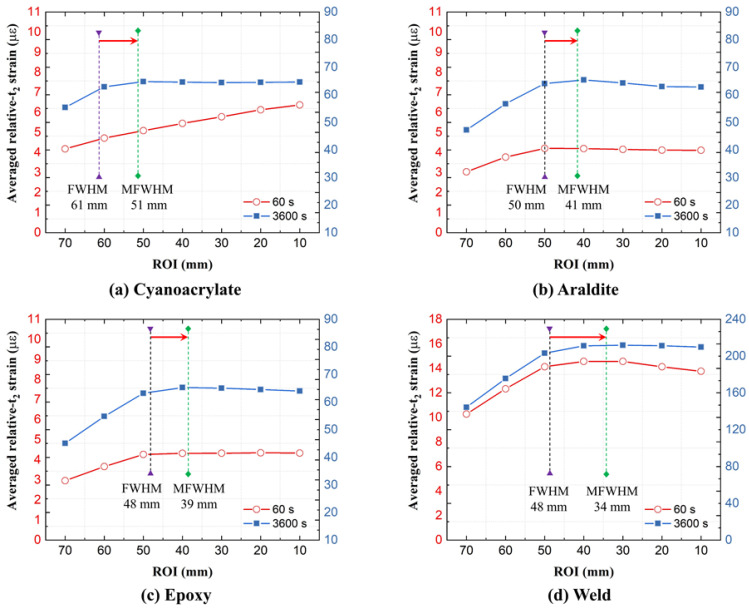
Average relative-*t*_2_ OF strains as a function of the region of interest (ROI) length on GF/Epoxy specimens.

**Figure 15:**
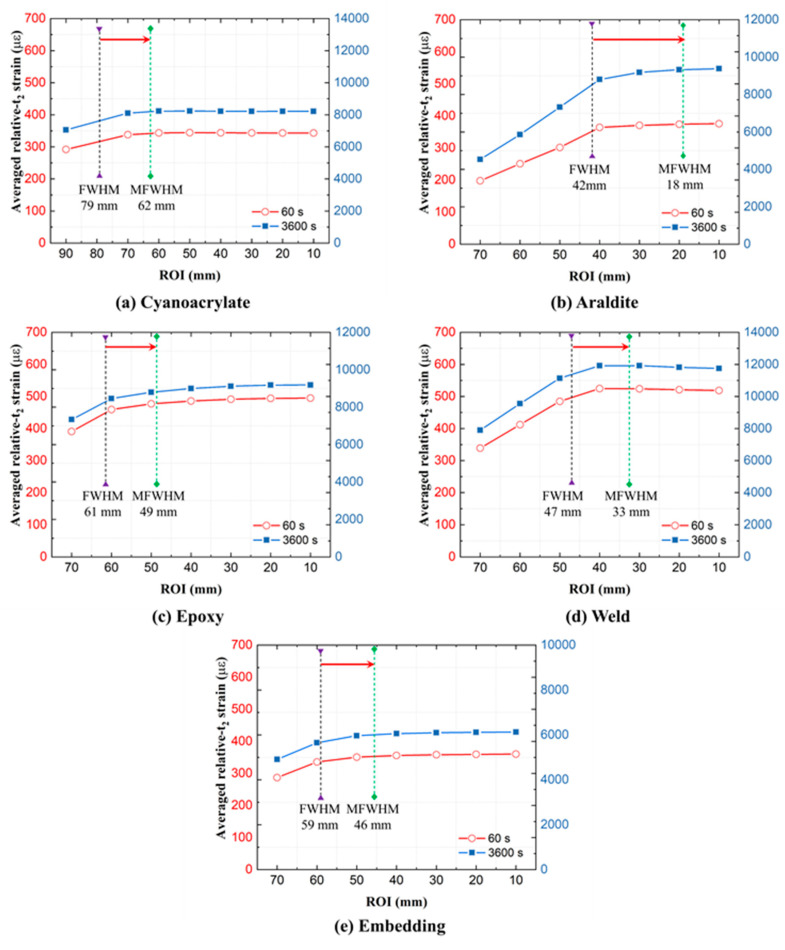
Average relative-*t*_2_ OF strains as a function of the region of interest (ROI) length on PA6 specimens.

In practical SHM applications, the strain value from the OF sensor is used to assess the strain state of the host component. However, disturbed strain regions at the ingress/egress of the optical fiber attachment have to be excluded from the strain analysis. These disturbed regions are present at every transition between a free and an attached or embedded optical fiber (including when OF passes through a void inside the structure). Only the middle of the fiber contains ROI suitable for interpreting substrate strains. Thus, especially for short OF attachment lengths, the selection of ROI (or alternatively, the ingress/egress lengths) becomes important for accurate strain analyses. The choice of ROI defines how much of the attachment ends are discarded. It filters out inaccurate ingress and egress regions of the attached OF. Throughout previous analyses, the ROI was defined as the central 40 mm for GF/Epoxy and PA6 specimens. This choice is hereby scrutinized. The influence of the ROI length on averaged relative-*t*_2_ OF strains is shown in Figure 14 for GF/Epoxy and in Figure 15 for PA6 substrates. Average strains at 60 and 3600 s were calculated using different ROIs. Average strains first increase with decreasing ROI, and then remain constant on a plateau when the disturbed ends become fully excluded. As evident, previously selected ROIs of 40 mm are positioned at the beginning of the plateaus and were indeed a good choice to achieve accurate average strain values for both the GF/Epoxy and PA6 specimens.

Disturbances in the ingress and egress regions are partially caused by averaging errors from the OBR post-processing, as discussed in Section 2.3. Experience with OBR strain measurements shows that steep strain gradients tend to produce more measurement noise and thereby also play a role in the size of these disturbed regions. The most accurate way to identify disturbed regions from experimental data is by parametric analysis, similar to Figure 14 and Figure 15. Alternatively, disturbed lengths *l* (Figure 3b) can be identified manually/visually directly from strain profiles. Using this manual approach, disturbed region lengths *l* were read from relative-t_2_ strain profiles at 3600 s as shown in Table 4. For GF/Epoxy, lengths *l* varied around 10 mm, when the gauge length (GL) for the virtual OBR sensor was selected as 10 mm. When the gauge length GL was set to 20 mm, the disturbed region lengths also doubled. For PA6, the disturbed region length *l* was much less predictable, typically exceeding the selected gauge length GL. The strain profiles of the PA6 specimens were inconsistent and fluctuations on the strain profiles make an accurate length *l* difficult to extract. It shows that the disturbed region length *l* cannot be simply defined equal to the OBR gauge length. Additionally, a more thorough analysis without human inspection would be preferred.

**Figure 16:**
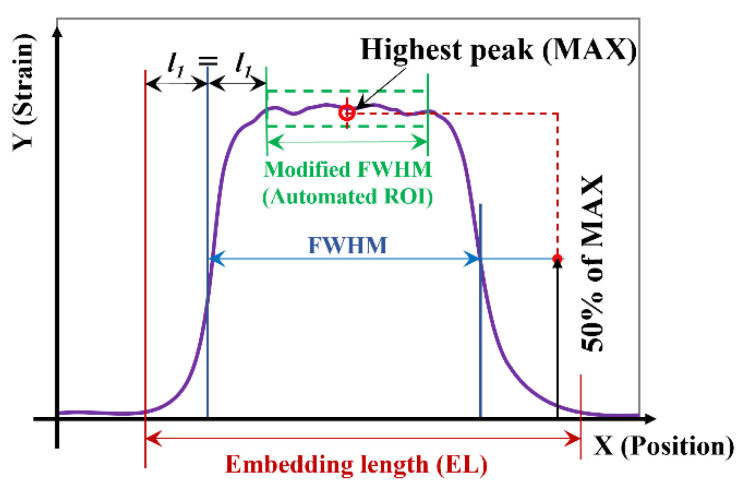
Automated ROI definition using regular and modified FWHM.

Automated ROI definition can be devised based on the full width half maximum (FWHM) concept for the spatial strain curve as shown in Figure 16. FWHM is the width of the strain curve measured between two strain points, which are at half of the maximum peak value. FWHM calculation is easy to automate. As shown in Figure 14 and Figure 15, a gap still exists between the FWHM-based ROI and the plateau of the strain–ROI curve. So, the FWHM, when used directly as ROI, is inaccurate. As described in Figure 16, the modified FWHM (MFWHM)-based automated ROI can be more accurate, defined by:

(2) $$ROI=EL-4\times l_{1}$$

where *EL* is the embedding length of DOFS and *l*_1_ is the gap between the *EL* and FWHM at one end of the curve. Hereby, *EL* is defined as the length of the strain curve measured between the two strain points where the strain first exceeds 10 µε. In this method, the length difference between the automated ROI (i.e., MFWHM) and the regular FWHM is assumed to be 2*l*_1_. As shown in Figure 14 and Figure 15, using MFWHM provides a fairly accurate alternative for the ROI selection.

#### 4.2.2. The Difference between OBR and Contact Extensometer Strains

**Figure 17:**
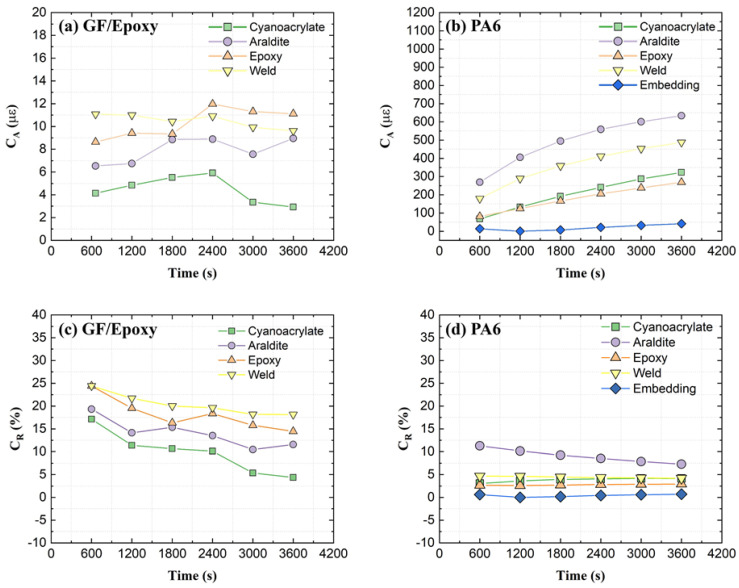
Difference coefficients *C*(*t*) between OBR and extensometer strains.

The strain measurement accuracy of the attached OF can be determined by comparing averaged OBR strains to contact extensometer strains along the same specimen length. The difference between OBR and extensometer strains can be defined by two sets of coefficients *C*(*t*):

(3) $$C_{A}(t)=\left|\varepsilon_{OBR}^{}(t)-\varepsilon_{EXT}^{}(t)\right|$$

(4) $$C_{R}(t)=\left|\frac{\varepsilon_{OBR}^{}(t)-\varepsilon_{EXT}^{}(t)}{\varepsilon_{EXT}^{}(t)}\right|\times 100\%$$

where coefficients *C_A_* and *C_R_* denote absolute and relative differences, respectively. Coefficient *C_R_* is similar to the strain transfer coefficient from the substrate to the OF, provided that the contact extensometer strain is equal to the substrate strain. Variables *ε_OBR_* (*t*) and *ε_EXT_* (*t*) are relative-*t*_2_ strains from the OBR and extensometer as shown in Figure 5 and Figure 7 at times *t* = 600, 1200, 1800, 2400, 3000, and 3600 s, respectively. The calculated coefficients *C*(*t*) for all attachments on the GF/Epoxy and PA6 substrates are shown in Figure 17.

For GF/Epoxy specimens, only very low absolute strain differences (ca. 3–12 µε) are reported in Figure 17a. Since Equation (3) is the numerator for Equation (4), and its value remains nearly constant while the denominator increases with creep, the relative difference *C_R_* as a consequence shows a decreasing trend for GF/Epoxy in Figure 17c. The low-viscosity ‘Cyanoacrylate’ attachment gave the smallest difference coefficients *C*, and thereby the best agreement between OBR and extensometer strains. The manually applied ‘Weld’ attachment, on the other hand, showed the largest difference between the OBR and extensometer strains.

For PA6 substrates, much higher absolute strain differences *C_A_* (between 13 and 650 µε) can be noted from Figure 17b. The absolute strain difference increases similar to the measured strains themselves, as seen in Figure 7. This causes a nearly constant relative strain difference *C_R_* as calculated in Figure 17d. The ‘Embedding’ attachment displays excellent agreement and nearly identical values from two strain measurement methods. Again, the manually applied ‘Araldite’ attachment gives the largest difference between the OBR and extensometer strains.

In summary, all relative differences *C_R_* between the extensometer and OBR strains are observed to either remain constant or decrease with time. The best agreement between the OBR and extensometer strains is achieved by the automated ‘Embedding’ and low-viscosity ‘Cyanoacrylate’ attachments. The worst agreement of strains is obtained for the manually controlled ‘Araldite’ and ‘Weld’ attachments.

## 5. Conclusions

1. As experimentally demonstrated, optical fiber sensors can be used to measure residual strains created by their own attachment process. The mechanisms of residual strain creation were briefly discussed. Correlations between residual strain and creep strains were observed for ‘Epoxy’ and ’Embedding’ attachment methods.
2. Creep strains up to 3% were measured from OFs fixed with five different attachment methods on three types of substrate specimens.
  - Unreinforced PA6 and GF/Epoxy substrates gave a satisfactory agreement between the optical fiber and contact extensometer strains. The relative difference between OF strains and contact extensometer strains either remained constant or converged towards more similar values over time.
  - Negligible creep strains of steel specimens were accurately measured only by OFs, as the contact extensometer displayed artificial warmup drift.
3. Problem areas for using DOFS over short attachment lengths are identified as follows.
  - Unreliable strain data occurs in the ingress and egress regions of the fiber.
  - Strain fluctuations along the OF length are caused by nonuniformities created in the fiber attachment process.
4. Optical fiber attachment methods were compared from the aspects of residual strains and creep strain development. The main takeaways from the experiments are summarized in Table 5. The best performing attachments were ‘Cyanoacrylate’ and ‘Embedding’. Concluding from these qualitative observations, an optimal optical fiber attachment method:
  - Is machine-controlled, e.g., utilizes an attachment process, such as 3-D printing, to achieve a uniform residual strain profile and a high strain transfer coefficient;
  - Uses a low-viscosity adhesive, such as cyanoacrylate, for the same reasons as previous;
  - Aims to minimize residual strains, e.g., by using room temperature curing or annealing.
5. Practical and easily automated approaches can be devised for defining the disturbed ingress/egress region lengths for strain measurement. For example, the modified FWHM approach gives fairly accurate estimations.

## Figures and Tables

**Table 1:** An overview of optical fiber attachment concepts.

Substrate	Attachment Method	Adhesives	Application Case
Concrete, steel, and timber	Surface mounting	Cyanoacrylate, epoxy, quartz glue, polyester	Strain, cracking, and vibration [2,3,4]
	Pre-embedded bar	Epoxy, silicone, rubber	Strain [5,6,7]
	Specialized optical cables	Epoxy	Creep strains and temperature [8]
Thermosets and thermoset composites	Surface mounting	Cyanoacrylate	Stiffness degradation and strain [9]
	Embedding	Epoxy resin	Impact damage [10,11]
Thermoplastics and thermoplastic composites	Surface mounting	Cyanoacrylate	Strain [12]
	Embedding (Hand-layup)	Inside the composite	Residual strains [13]
	Embedding (Hot-pressing)	Partially fixed with epoxy	Relaxation [14]
	In situ embedding (3-D printing)	Inside the polymer	Residual strains and defects [15]

**Table 2:** Dogbone specimen dimensions (mm), after ASTM E8 [18] and ASTM D638 [19], with reference to Figure 1.

Dimensions	PA6	GF/Epoxy	Steel
GL—Gauge length	50	50	25
GW—Width	13	13	6
T—Thickness	6.4	7	3
R—Radius of fillet	76	76	6
L—Overall length	165	165	100
A—Length of reduced parallel section	57	57	32
B—Distance between grips	115	115	40
W—Width of grip section	19	19	10
OF L—Attached optical fiber length	50	50	25
EXT L—Extensometer gauge length	50	50	25

**Table 3:** Investigated optical fiber attachment methods.

Attachment Denotation	Shorthand Name	Method	Details ^1^
a	‘Cyanoacrylate’	Cyanoacrylate adhesive	Standard adhesive for strain gauges.
b	‘Araldite’	Araldite Rapid adhesive	Two component rapid curing epoxy.
c	‘Epoxy’	Epoxy film adhesive	Adhesive film (Gurit SA 80) is placed over the OF, and cured for 12 h at 80 °C under vacuum.
d	‘Weld’	OF is manually fused/glued on the substrate by a filament of thermoplastic material	A PA6 filament (1.75 mm, natural, Ultrafuse) is melted and extruded with a Leister Triac hot-air tool.
e	‘Embedding’	The OF is 3-D printed under a cuboid volume (64 mm × 10 mm × 0.4 mm) embedding it directly on the surface of the PA6 specimen.	PA6 (1.75 mm filament, natural, Ultrafuse).

^1^ Room temperature varied between 19 and 25 °C and relative humidity between 15 and 35% during the attachment and testing procedures.

**Table 4:** The influence of the OBR virtual gauge length (GL) on disturbed region lengths *l*, at *t* = 3600 s (all dimension in mm).

Specimen, Attachment Method	Actual OF Attachment Length	Disturbed Ingress Region *l*		Disturbed Egress Region *l*	
		OBR GL = 10	OBR GL = 20	OBR GL = 10	OBR GL = 20
GF/Epoxy, Cyanoacrylate	70	9.0	17.5	8.0	19.0
GF/Epoxy, Araldite	51	9.5	16.5	9.0	16.5
GF/Epoxy, Epoxy	53	8.0	17.5	11.5	22.0
GF/Epoxy, Weld	55	14.5	16.5	9.0	19.0
PA6, Cyanoacrylate	77	22.0	29.5	18.5	30.5
PA6, Araldite	54	15.0	23.5	28.0	30.5
PA6, Epoxy	58	11.0	25.0	10.0	24.0
PA6, Weld	59	16.0	16.5	14.0	16.5
PA6, Embedding	64	24.0	31.5	13.0	25.0

**Table 5:** Qualitative comparison of DOFS attachment methods on three different substrates.

Attachment Method	Substrate	Residual Strain (Figure 10)	Creep Strain Variability (Figure 13)	Creep Strain Accuracy (Figure 17)	Attachment Process
‘Cyanoacrylate’	GF/Epoxy	Low/Nonuniform	Low	High	Manual
	PA6	Low/Nonuniform	Low	Medium	Manual
	Steel	Low/Nonuniform	-	-	Manual
‘Araldite’	GF/Epoxy	Low/Nonuniform	High	Medium	Manual
	PA6	Low/Nonuniform	High	Low	Manual
	Steel	Low/Nonuniform	-	-	Manual
‘Epoxy’	GF/Epoxy	Medium/Uniform	Low	Low	Manual
	PA6	High/Uniform	High	Medium	Manual
	Steel	Medium/Uniform	-	-	Manual
‘Weld’	GF/Epoxy	Medium/Nonuniform	Low	Low	Manual
	PA6	High/Nonuniform	High	Medium	Manual
	Steel	Medium/Nonuniform	-	-	Manual
‘Embedding’	GF/Epoxy	-	-	-	-
	PA6	High/Uniform	Low	High	Automated
	Steel	-	-	-	-

**Figure A1:**
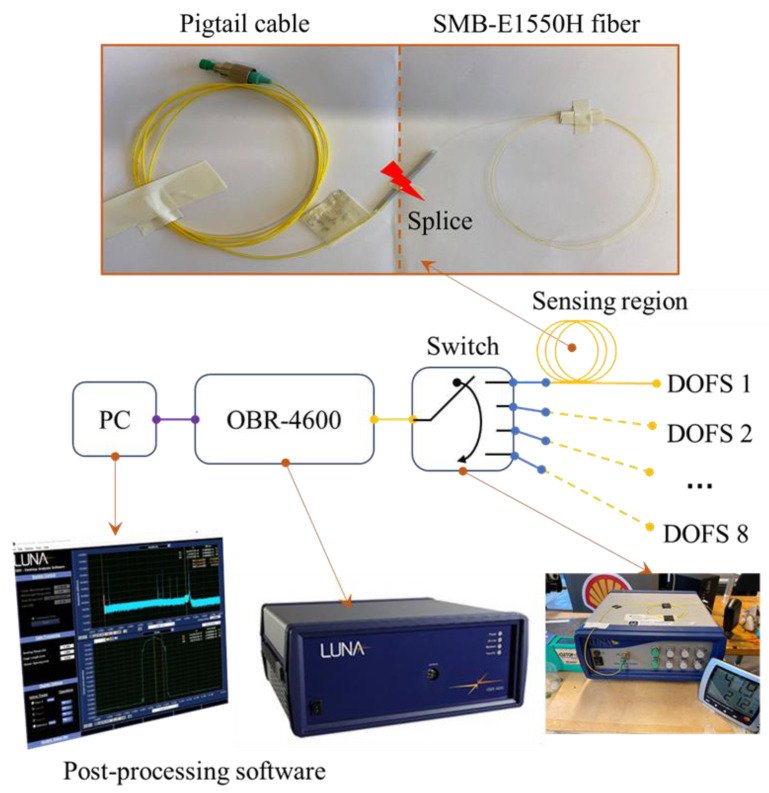
Distributed strain sensing system.

**Figure A2:**
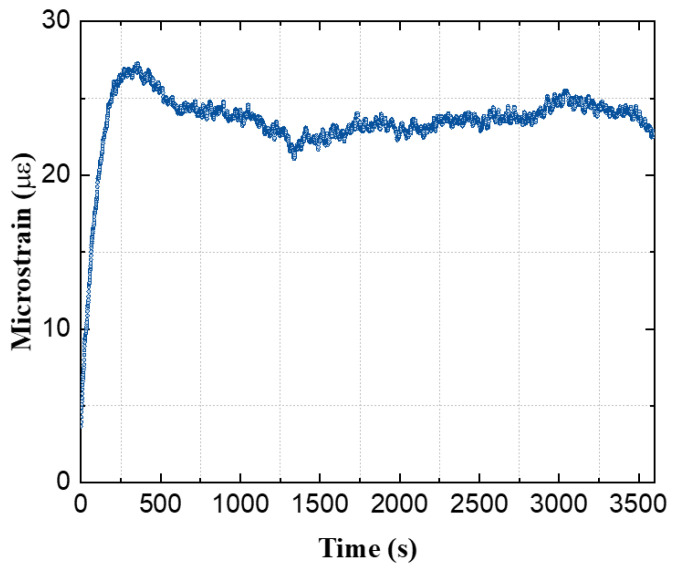
Strain from the contact extensometer on a steel specimen (unloaded, hanging by a hook) at room temperature.

**Figure A3:**
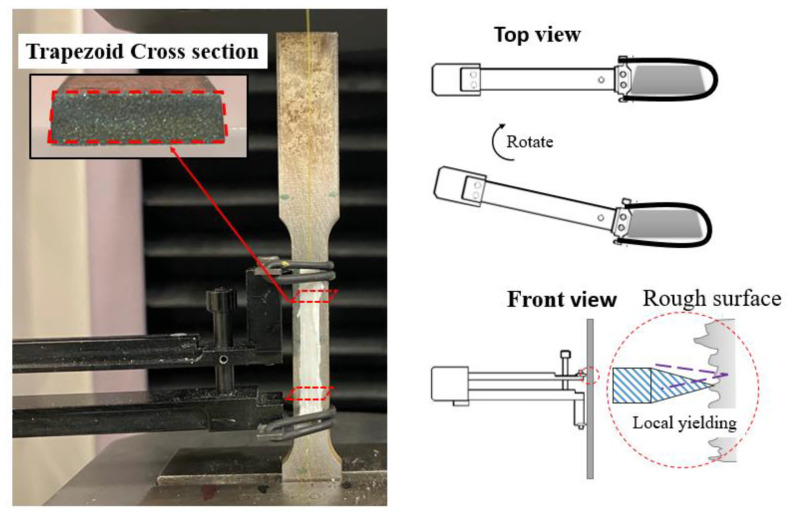
Other possible causes for measurement errors for the contact extensometer.

## Data Availability

Regarding raw data availability, please contact the authors.

## Appendix A. Distributed Strain Sensing System

As shown in Figure A1, the distributed strain sensing system consists of a distributed optical fiber sensor (DOFS), a PC, an optical fiber switch, and an interrogator device. The DOFS is obtained by splicing a data transfer cable to a sensing optical fiber. The sensing fiber section is a 1-m-long single mode fiber with an operating wavelength of 1550 nm. The fiber SMB-E1550H was purchased from OFS Fitel. It is a silica/silica/polyimide fiber with a core diameter of 6.5 μm, a cladding diameter of 125 μm, and a coating diameter of 155 μm. The data cable is 1.5 m long, reinforced with a rubber jacket, and ends with a pigtail. All DOFS are first connected to an optical fiber switch and then to the Optical Backscattering Reflectometer OBR 4600 from Luna Instruments. OBR 4600 measures Rayleigh backscatter over the full length of the DOFS. Detailed technical parameters of the OBR 4600 device are listed in Table A1. During the measurement procedure, a laser source sends the incident light through the OF, and subsequently Rayleigh backscattering occurs along the entire fiber. Changes on the fiber, such as strain and temperature, induce a frequency shift to the reflected light spectrum. These Rayleigh backscattering spectral shifts are measured and scaled to give distributed temperature or strain measurements with a high spatial resolution.

**Table A1:** Specifications of the OBR 4600 device.

Laser	Tunable Laser Source (TLS)
Wavelength Range	1525–1610 nm
Internal laser module maximum rated output power	10.0 mW
Standard mode	30 m/70 m
Extended mode	2000 m
Scan time (30 m mode)	3 s
Sensitivity	−130 dB
Dynamic range	80 dB
Spatial resolution (30 m mode)	20 μm
Strain resolution	±1.0 µϵ
Temperature resolution	±0.1 °C

## Appendix B. Uncertainties of Contact Extensometer Strains

A small load was applied on steel specimens. Uniform stress in the gauge region of the steel dogbone was estimated as 46 MPa, much smaller than its yield strength of 250 MPa. Thus, the measurement should display linear elastic behavior and no rise in strain during one hour of room temperature creep. As evident from Figure 9, all strain–time curves measured by the OBR fluctuate around zero. The extensometer, however, showed increasing strains before ca. 1500 s, which then remained flat after 1800 s. This deviation of extensometer strains from linear elastic behavior (i.e., near-zero strain values) was unexpected.

One possible reason is the warmup drift [21]. When the extensometer is first powered, the flow of current generates heat. This heat produces a small artificial strain drift. After the temperature of the extensometer stabilizes, the warmup drift will also stabilize. The process is affected by time, excitation voltage, and room temperature. The warmup drift effect was replicated for the used extensometer in additional proof-of-concept experiments, e.g., as shown in Figure A2. This warmup drift also probably contributed to the small but consistent strain difference between the OBR and extensometer strains for the GF/Epoxy specimens (Figure 4 and Figure 5). The presence of warmup drift is much less consequential for PA6 specimens since their strain values in Figure 6 and Figure 7 were orders of magnitude higher.

Figure A3 suggests a few other reasons that can potentially induce measurement errors for the contact extensometer. The trapezoid cross-sectional profile of the steel specimen was created by water jet cutting. The knife-edges were positioned on the canted and rough edge surfaces and then fixed by rubber bands. During the test, the extensometer might rotate slightly on the edge of the specimen. Local yielding may also happen at the contact points of the knife-edges. Attaching the contact extensometer knives on the canted and narrow edges of the steel specimens is not the best measurement practice.

A stringent reasoning of why small creep strains were measured by the contact extensometer, as witnessed in in Figure 9, cannot unfortunately be provided after the testing was finished. Warmup drift is likely the biggest contributing factor. Possible misalignment and contact imperfections at the knife-edges can also contribute to measurement uncertainties. However, these effects should rather reduce the measured strain values. For the steel specimens of this creep experiment, the OFs clearly provide more accurate strain data (in agreement with expectation) compared to the contact extensometer strains.
